# Avian Coronavirus Infectious Bronchitis Virus Activates Mitochondria-Mediated Apoptosis Pathway and Affects Viral Replication by Inducing Reactive Oxygen Species Production in Chicken HD11 Cells

**DOI:** 10.3390/biology13070491

**Published:** 2024-07-01

**Authors:** Xiaoxiao Han, Yuan Huang, Junli Hao

**Affiliations:** School of Bioscience and Technology, Chengdu Medical College, Chengdu 610500, China; huangyuan4088@163.com (Y.H.); haojunli00@163.com (J.H.)

**Keywords:** infectious bronchitis virus, reactive oxygen species, apoptosis, N protein, chicken HD11 cells

## Abstract

**Simple Summary:**

Avian infectious bronchitis is an important infectious disease that causes heavy losses in the poultry industry. Vaccination is an effective preventive measure, but the ability of infectious bronchitis virus (IBV) to mutate reduces the vaccine’s protective effect. Therefore, it is quite important to study the pathogenic mechanism of IBV. In this study, we found that IBV infection leads to ROS accumulation in chicken macrophage HD11 cells. The oxidative stress further induced cell apoptosis mediated by mitochondria-mediated apoptotic signaling. In addition, IBV replication was essential to ROS accumulation, and IBV proliferation decreased when ROS activation was blocked. IBV nucleocapsid (N) protein can cause ROS accumulation and cell apoptosis. Our findings may provide insights into the interaction between IBV and its host and uncover the pathogenesis of IBV infection.

**Abstract:**

Infectious bronchitis virus (IBV), a coronavirus that causes severe respiratory and gastrointestinal illness in poultry, leads to substantial economic losses. According to earlier research, IBV infection causes chicken macrophage HD11 cells to undergo cell apoptosis. Reactive oxygen species (ROS) and the IBV-activated intrinsic apoptotic signaling pathway were examined in this work. The findings demonstrate that IBV infection causes ROS to accumulate. Moreover, IBV infection decreased the mitochondrial transmembrane potential in HD11 cells, which could be blocked by ROS antioxidants (PDTC and NAC). The two antioxidants significantly affected the expression of Bcl-2 and Bax and further inhibited the activation of caspase-3 and apoptosis in HD11 cells. Additionally, IBV replication was decreased by blocking ROS accumulation. Pretreating HD11 cells with ammonium chloride (NH_4_Cl) prevented IBV from entering the cells and reduced the oxidative stress which IBV causes. The ability to accumulate ROS was also lost in UV-inactivated IBV. The IBV N protein induces cell apoptosis through the activation of ROS. These findings provide an explanation for the processes of IBV infection in immune cells by indicating that IBV-induced ROS generation triggers cell apoptosis in HD11 cells.

## 1. Introduction

Avian infectious bronchitis (IB) is a serious disease that spreads quickly. It is characterized by respiratory disease and nephritis and can cause a reduction in both egg production and egg quality in laying flocks [1,2]. IBV is a pathogen that belongs to the Coronaviridae family and has a positive-sense, single-stranded RNA genome [3]. It is present worldwide and causes economic losses for the poultry industries in many countries. Vaccination has been considered the most effective method for IBV control [4]. However, because of the continuous emergence of new mutant viruses, the current vaccines have proved to be inadequate [5,6]. Thus, studying the signaling pathway and regulation mechanism of IBV in chicken-derived cells is the basis for prevention and control and will help to reveal the pathogenesis and provide a theoretical basis for vaccine research. Previous studies on the pathogenesis of IBV mainly focused on primary cells [7]. However, there is less information about the signaling pathways induced by IBV infection in immune cells. Even we have previously shown that IBV causes apoptosis in HD11 cells [8]. However, it is currently unclear how IBV infection activates the upstream apoptotic signaling.

Oxidative stress, which can be caused through the host’s immune system or by viruses directly, is a plausible significant pathogenic factor in numerous viral infections [9,10,11,12]. Reactive oxygen species (ROS) production is frequently mentioned as an oxidative stress cause [13]. Most vertebrate cells produce ROS, which are typically thought of as dangerous metabolites and function as signaling molecules, primarily in the mitochondria. Viral infections, chemotherapeutic medicines, and ionizing radiation are just a few of the stressors that can cause ROS [14]. In addition to killing the target cells, ROS also damage surrounding cells’ DNA and suppress cellular antioxidant defenses. Many biological functions, including cell apoptosis, inflammation, and proliferation, are regulated by ROS [15].

The regulation of several pathogenic processes, including cellular apoptosis, is affected by ROS. Moreover, growing evidence suggests that ROS accumulation is also relevant to virus-induced apoptosis [16,17]. When cells undergo apoptosis, excessive ROS are damaging to cells. The cell apoptosis induced by the virus can be enhanced by ROS accumulation through disrupting mitochondrial function, activating mitochondrial oxidative channels, and releasing mitochondrial cytochrome C into the cytosol [18]. Animal viral infections, like porcine parvovirus (PPV) and transmissible gastroenteritis virus (TGEV), may induce host cell apoptosis by accumulating ROS [19,20]. In our previous studies, IBV infection induced cell apoptosis through either intrinsic or extrinsic pathways [8]. It has been shown that the activation of mitochondria, which is mediated by ROS, is linked with intrinsic apoptotic pathway initiation. The relationship between ROS and IBV-induced apoptosis remains uncertain though. In addition, whether IBV nucleocapsid (N) protein could affect ROS accumulation and the associated cell apoptosis is not clear.

In this research, we used HD11 cells as a viral infection model to examine whether IBV infection could lead to ROS production. Moreover, IBV infection can also decrease the mitochondrial transmembrane potential (Δψm). Ammonium pyrrolidine dithiocarbamate (PDTC) and N-acetyl-L-cysteine (NAC) are two scavengers that greatly reduce apoptosis activation by reducing IBV-induced Bcl-2 reduction, an increase in Bax, and caspase-3 activation. In IBV-infected cells, ROS accumulation can also have an impact on viral replication. IBV N protein, when expressed within cells, can cause ROS activation and apoptosis in HD11 cells

## 2. Materials and Methods

### 2.1. Cell Culture and Virus Infection

Chicken macrophage HD11 cells were cultivated in Dulbecco’s modified Eagle’s medium (DMEM) (HyClone, Logan, UT, USA), enriched with 10% fetal bovine serum (FBS) (Gemini Bio-Products, West Sacramento, CA, USA), 100 U/mL penicillin, and 100 μg/mL streptomycin at a pH of 7.2, and maintained at 37 °C with 5% CO_2_. South China Agricultural University Professor Ding-Xiang Liu donated the IBV Beaudette strain (GenBank: DQ001339). HD11 cells were seeded and grown in 6 well plates for 24 h before being treated with IBV at a multiplicity of infection (MOI) of 10, followed by 5% CO_2_ incubation at 37 °C for 2 h. After a PBS wash (HyClone, Logan, UT, USA), the cells were supplemented at 37 °C in DMEM with 2% FBS. By utilizing the Reed and Muench method with 50% tissue culture infective doses (TCID_50_), viral titers were identified.

### 2.2. Fluorescence Microscopy Observation of ROS Generation

An ROS assay kit was utilized to identify ROS (Beyotime, Shanghai, China). ROS levels were measured in IBV-infected HD11 cells using DCFH-DA and then observed under fluorescence microscopy to research the upstream mechanism of IBV-induced cell apoptosis [21]. The cells were cultured in 24 well tissue culture plates and then infected with IBV at a multiplicity of infection (MOI) of 10 at different times. Subsequently, the cells were subjected to 30 min of incubation at 37 °C in the absence of light following 10 μM 2′,7′-dichlorodihydrofluorescein (DCFH-DA) treatment. Fluorescence microscopy was used to observe the cells (Olympus IX71, Tokyo, Japan).

### 2.3. ROS Determination by Flow Cytometry

Cells with IBV infection and a mock infection were collected at the times specified. The cells were subjected to a half-hour incubation period at 37 °C in the absence of light, during which they were exposed to 10 μM DCFH-DA. Following two DMEM washes devoid of FBS, the cells were examined by flow cytometry analysis.

### 2.4. Measurement of Mitochondrial Transmembrane Potential (Δψm)

The Δψm was assessed utilizing a JC-1 Mitochondrial Potential Detection Kit (Beyotime, Shanghai, China) as per the manufacturer’s guidelines. In summary, cells were collected at the specified time, stained at room temperature with JC-1 in PBS for 15 min in the dark, and then evaluated by utilizing flow cytometry. At 530 nm and 488 nm, the fluorescence signal was measured.

### 2.5. Quantitative Real-Time PCR (qRT-PCR) Analysis

The total RNA was isolated utilizing TRIzol agent (Invitrogen, Carlsbad, CA, USA), and cDNA was synthesized from each RNA sample utilizing a PrimeScriptTM RT Reagent Kit (Takara, Dalian, China). For qRT-PCR analyses, the cDNA was utilized. Table 1 shows the primer sets for the genes that regulate apoptosis [22]. The qRT-PCR experiment’s 25 μL mixture consisted of 2 μL of cDNA and 12.5 μL of SYBR Premix Ex TaqTM II (Takara, Dalian, China), with 1 μL each of forward and reverse primers and 8.5 μL of RNAase-free waters. Using a thermal cycler (Bio-Rad IQ5, Hercules, CA, USA), the reaction conditions used were as previously described in [8]. As a reference gene, β-actin was used. By using 2^−ΔΔCT^, the expression fold changes were computed [23].

### 2.6. Caspase-3 Activity Assay

A colorimetric assay kit (KeyGEN Biotech, Nanjing, China) was utilized to explore whether caspase-3 was active. The cells were treated with lysis buffer, and BCA protein assay reagent (Vazyme, Nanjing, China) was used to measure the protein concentrations. The protein (200 μg/sample) was then incubated at 37 °C with caspase-3 in each sample for 4 h. At 405 nm, the samples were detected using a micro-plate reader (Bio-Rad 680, Hercules, CA, USA).

### 2.7. Apoptotic Rate Measurement

An Annexin V-FITC apoptosis detection kit (Absin, Shanghai, China) was utilized to calculate the cell’s percentage of occurring apoptosis to determine the apoptotic rate. IBV of 10 MOI was used to infect HD11 cells cultivated in 6 well tissue culture plates. The collected cells were washed three times with PBS at set times. Following 500 g of centrifugation for 5 min, the cells were managed as per the manufacturer’s guidelines. Within an hour, the cells were assessed by utilizing a flow cytometer (BD Biosciences, San Jose, CA, USA).

### 2.8. Plasmid Construction and Cell Transfection

The IBV N sequence was amplified by the cDNA extracted from the IBV Beaudette strain and subsequently cloned into pcDNA3.1-Flag empty vectors. Plasmids were extracted using the endotoxin-free plasmid extraction kit (Tiangen, Beijing, China). The in vitro expression of the IBV N protein was assessed through transient expression experiments using HD11 cells. The HD11 cells were plated in 6 well plates at a density of 5 × 10^5^ cells/mL and incubated for 12 h. Transfection was carried out using the Lipofectamine 8000 system (Beyotime, Shanghai, China), with cells transfected with either pcDNA3.1-Flag-N or pcDNA3.1-Flag (as an empty vector) for 48 h. The transfection efficiency was directly observed under fluorescence microscopy and further confirmed using qRT-PCR to detect the IBV-N gene mRNA.

### 2.9. Indirect Immunofluorescence Assay (IFA)

The HD11 cells were transfected with pcDNA3.1-Flag-N. After 48 h, the cells were immobilized with 4% paraformaldehyde for 30 min. Flag tag mouse monoclonal antibody (Beyotime, Shanghai, China) was added, followed by incubation for 1 h at 37 °C. Next, the cells were treated with a fluorescein (FITC)-conjugated affinipure goat anti-mouse IgG (H+L) (Sanying Biotech, Wuhan, China) for 45 min at 37 °C. The specimens were viewed with a fluorescence microscope (Olympus IX71, Tokyo, Japan) with the appropriate excitation and emission wavelengths for FITC (492 nm and 520 nm, respectively).

### 2.10. Statistical Analysis

Three separate trials were conducted in duplicate, and all data were presented as the mean ± SEM. Statistical analysis was performed using GraphPad Prism (V5). A Student’s unpaired t-test was used to assess the statistical significance between two groups, while ANOVA was employed to compare the means among three or more groups. Statistical significance was determined by *p* values less than 0.05 and 0.01, which were considered significant and highly significant, respectively.

## 3. Results

### 3.1. The Increase in ROS Was Induced by IBV Infection

**Figure 1:**
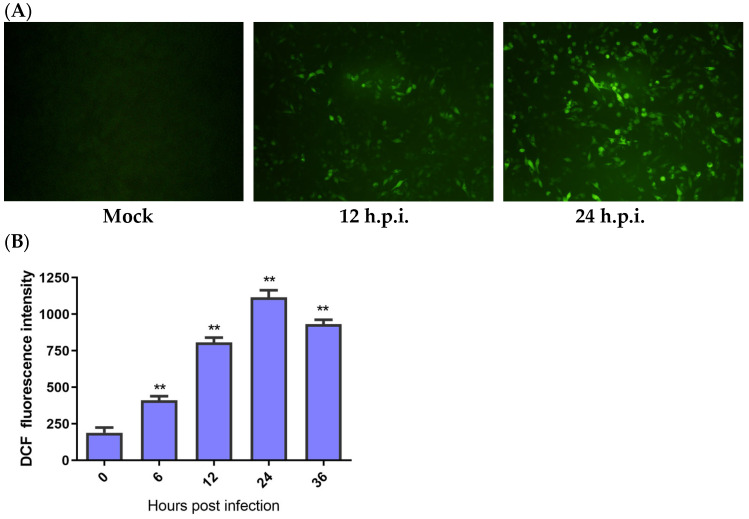
IBV infection-induced ROS production. HD11 cells were infected with IBV at an MOI of 10 at different times and then incubated with 10 μM DCFH-DA at 37 °C for 1 h in the dark. (**A**) The ROS productions observed under a fluorescence microscope. (**B**) The levels of ROS for each indicated time calculated by the fluorescence degree in IBV-infected cells while subtracting the fluorescence degree in the mock infected cells. Data are shown as the mean ± SEM. ** *p* < 0.01 versus control group (0 h).

According to the results, fluorescence in the HD11 cells was first detected at 12 h post-infection (h.p.i.), and it greatly increased at 24 h.p.i. (Figure 1A). A quantitative test of ROS was performed in the IBV-infected HD11 cells by using flow cytometry to further identify ROS production. The cells infected with IBV produced significantly more ROS at 6 h.p.i. than the mock infected cells. The fluorescence values of ROS continuously increased until 24 h.p.i. and then decreased at 36 h.p.i. (Figure 1B). These findings indicate that IBV could increase ROS levels in HD11 cells.

### 3.2. Mitochondrial Membrane Potential Was Decreased by IBV Infection

**Figure 2:**
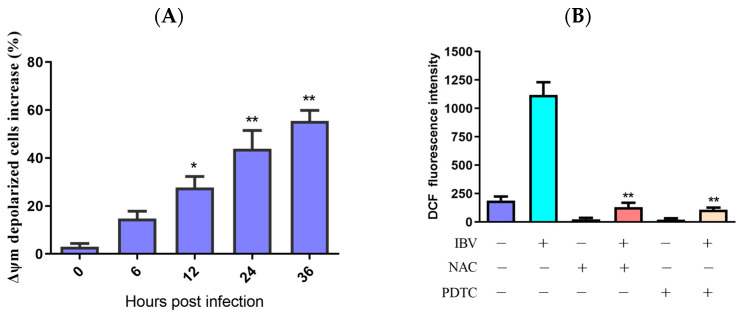

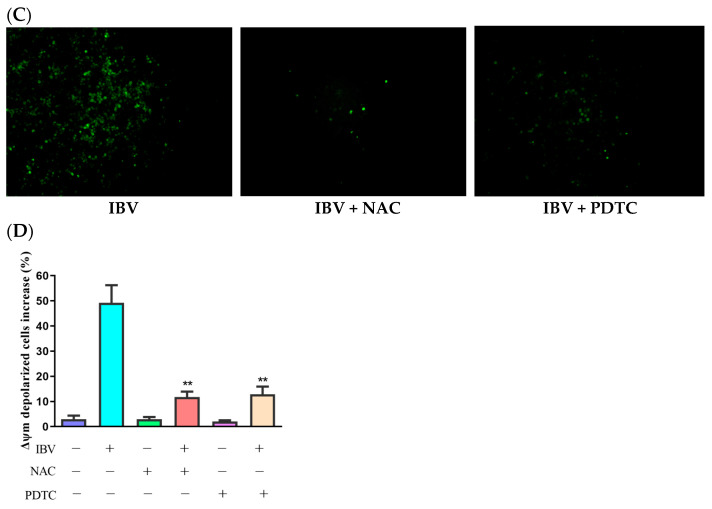
Mitochondrial membrane potential was reduced by ROS accumulation. (**A**) HD11 cells infected with 10 MOI of IBV at different times and stained with JC-1 for 15 min at room temperature. The increases in Δψm–depolarized cells were analyzed using flow cytometry. Data are shown as the mean ± SEM. * *p* < 0.05 versus control group (0 h). ** *p* < 0.01 versus control group (0 h). (**B**) HD11 cells were infected with IBV for 30 h in the presence or absence of NAC (10 mM) or PDTC (10 μM), and the ROS productions were analyzed using flow cytometry. Data are shown as the mean ± SEM. ** *p* < 0.01 versus IBV infection without antioxidants. (**C**) ROS productions observed under a fluorescence microscope. (**D**) The Δψm was analyzed using flow cytometry. Data are shown as the mean ± SEM. ** *p* < 0.01 versus IBV infection without antioxidants.

ROS might activate the mitochondrial apoptotic pathway. As Δψm collapses, the mitochondrial apoptotic signaling cascade is activated. To assess IBV infection’s impact on Δψm, we measured the Δψm of IBV-infected and mock infected HD11 cells while using JC-1 as a fluorescence probe via flow cytometry. As demonstrated in Figure 2A, the Δψm-depolarized cell percentage rose by 11.83% at 6 h.p.i., with an escalating degree of depolarization over time. To further examine the ROS influence on Δψm, we employed two distinct ROS antioxidants, PDTC (10 μM) and NAC (10 mM) (Beyotime Biotech, Shanghai, China), which did not show cytotoxicity at the used concentrations and were used to suppress ROS generation in the IBV-infected HD11 cells. The findings revealed that PDTC and NAC can both inhibit the production of ROS (Figure 2B,C). In addition, ROS scavengers can also reduce the Δψm of IBV-infected cells (Figure 2D). These findings propose that ROS may lower the Δψm during IBV infection.

### 3.3. The Function of ROS in Virus-Induced Intrinsic Apoptosis

**Figure 3:**
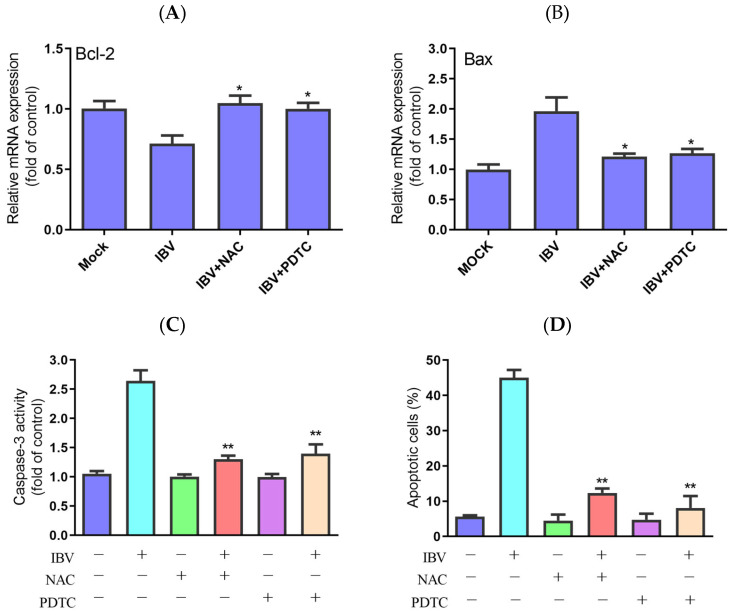
Effects of NAC and PDTC on intrinsic apoptosis. The cells were infected with IBV for 30 h in the presence or absence of NAC or PDTC. (**A**,**B**) The mRNA expression of Bcl-2 and Bax, analyzed using qRT-PCR. Data are shown as the mean ± SEM. * *p* < 0.05 versus IBV infection without antioxidants. (**C**) Caspase-3 activity detected by a colorimetric assay kit. Data are shown as the mean ± SEM. ** *p* < 0.01 versus IBV infection without antioxidants. (**D**) The apoptotic rate, analyzed using flow cytometry. Data are shown as the mean ± SEM. ** *p* < 0.01 versus IBV infection without antioxidants.

Generally, the intrinsic apoptosis upon virus infection is regulated by the mitochondria pathway. In addition, excessive ROS can cause damage to mitochondrial DNA and permeabilization of the mitochondrial outer membrane [13]. To better understand ROS accumulation’s roles in IBV-induced apoptosis, we evaluated the impact of PDTC and NAC on the expression of Bcl-2 and Bcl-2-associated X protein (Bax) in IBV-infected HD11 cells. In contrast to IBV infection alone, the treatment with two antioxidants clearly prevented the loss of Bcl-2 expression and a rise in Bax expression, as shown in Figure 3A,B. Additionally, both PDTC and NAC significantly reduced caspase-3 activity in the IBV-infected cells compared with the cells not treated with the two inhibitors (Figure 3C). At last, treatment with PDTC and NAC greatly reduced the apoptotic rate in the IBV-infected HD11 cells (Figure 3D). These findings indicate that ROS are crucial to the IBV-induced apoptosis process.

### 3.4. Interaction between Virus Replication and ROS Accumulation

**Figure 4:**
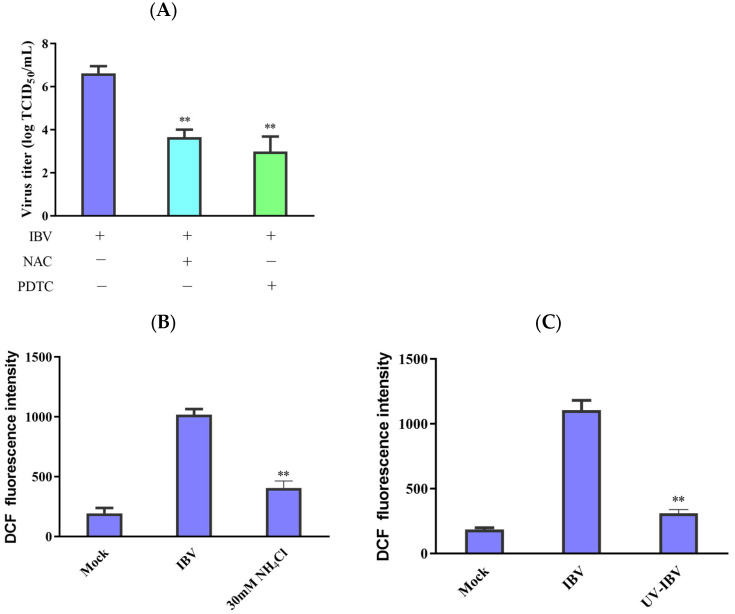
Interplay between ROS accumulation and virus replication. (**A**) Following incubation with antioxidants for 1 h, the cells were infected with IBV for 30 h. After pretreatment, virus titers were revealed in terms of lgTCID_50_/mL. The data are shown as mean ± SEM. ** *p* < 0.01 versus IBV infection alone. (**B**) The 30 μM concentration of NH_4_Cl was used to incubate the HD11 cells for 2 h before infection. Then, the cells were infected with IBV for 30 h. ROS production was analyzed using flow cytometry. The data are shown as mean ± SEM. ** *p* < 0.01 versus cells infected without NH_4_Cl. (**C**) UV germicidal light was utilized to inactivate IBV for 30 min. UV-inactivated virus infected HD11 cells for 30 h, and then ROS production was analyzed using flow cytometry. The data are shown as mean ± SEM. ** *p* < 0.01 versus UV-untreated IBV.

TCID_50_ determined the untreated and antioxidant-treated cell viability and virus titers to ascertain whether ROS has a significant function in viral replication inhibition. The findings demonstrate that both PDTC and NAC could reduce the IBV titers (Figure 4A). NH_4_Cl can inhibit endosomal acidification and inhibit the release of viruses [8,20]. IBV infection reduced the production of ROS in the NH_4_Cl-treated cells compared with the untreated cells (Figure 4B), and UV-inactivated IBV also lost its capacity to enhance ROS production (Figure 4C). Our data imply that ROS formation in IBV-infected HD11 cells depends on viral replication and virions entering the cells.

### 3.5. Expression of IBV-N Gene Induces ROS Accumulation and Apoptosis

**Figure 5:**
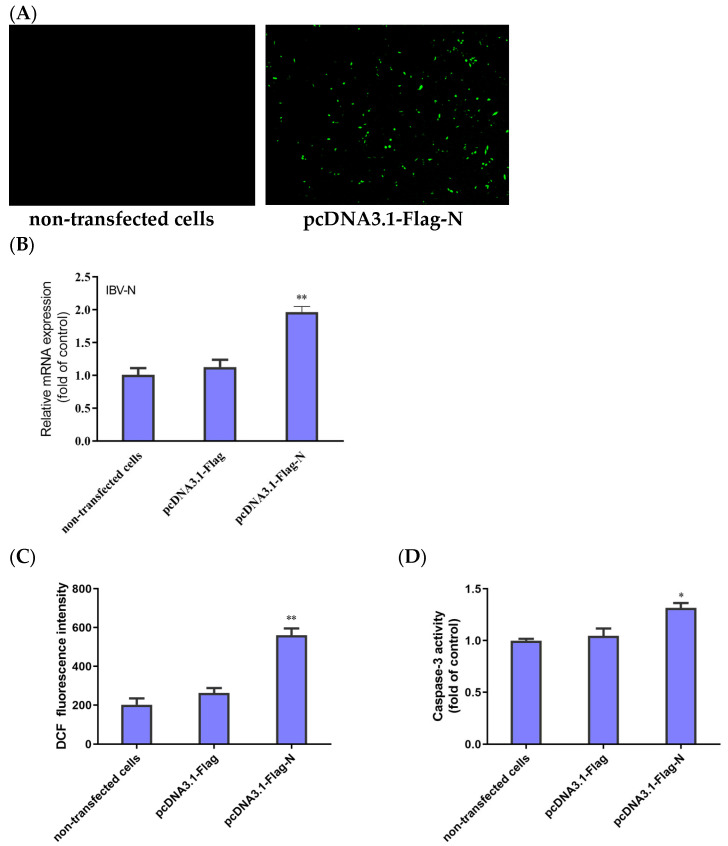
Expression of IBV-N gene in HD11 cells and its effect on ROS accumulation and apoptosis. (**A**) Fluorescence microscopic observation of HD11 cells at 48 h after transfection with pcDNA3.1-Flag-N and non-transfected cells. (**B**) HD11 cells transfected with pcDNA3.1-Flag-N or pcDNA3.1-Flag and non-transfected cells collected at 48 h. The relative expression of the IBV N gene was determined by qRT-PCR, using β-actin as the reference. The data are shown as the mean ± SEM. ** p < 0.01 versus the cells transfected with pcDNA3.1-Flag. (**C**) ROS productions were analyzed using flow cytometry. The data are shown as the mean ± SEM. ** p < 0.01 versus the cells transfected with pcDNA3.1-Flag. (**D**) Caspase-3 activity was detected with a colorimetric assay kit. Data are shown as the mean ± SEM. * *p* < 0.05 versus the cells transfected with pcDNA3.1-Flag.

The HD11 cells were transfected with pcDNA3.1-Flag-N, and the transfection efficiency was directly observed under fluorescence microscopy and further confirmed by qRT-PCR to detect IBV-N gene mRNA. After 48 h of transfection, the fluorescence plasmid could be detected with an IFA test to identify the successful transfection (Figure 5A). Compared with the blank control group (non-transfected cells) and negative control group (transfected with pcDNA3.1 plasmid), the qRT-PCR results show that the mRNA levels of the N gene were obviously upregulated in the HD11 cells transfected with pcDNA3.1-Flag-N (Figure 5B). These results suggest that the IBV-N gene is expressed in HD11 cells.

To examine whether ROS accumulation and apoptosis were initiated in the cells expressing the IBV-N gene, the HD11 cells transfected with pcDNA3.1-Flag-N or pcDNA3.1-Flag and the non-transfected cells were subjected to testing for the fluorescence values of ROS and the activity of caspase-3. As shown in Figure 5C, the cells transfected with pcDNA3.1-Flag-N produced significantly more ROS than the negative control group. Moreover, the activity of caspase-3 significantly increased in the cells transfected pcDNA3.1-Flag-N compared with the cells transfected with pcDNA3.1-Flag (Figure 5D). Taken together, the results demonstrate that the IBV N protein triggered the accumulation of ROS, leading to cell apoptosis.

## 4. Discussion

Apoptosis is autonomous and organized cell death controlled by genes which maintain homeostasis, and it is essential to the pathogenic process of viral infection. Many factors induce cell apoptosis, and ROS are crucial to the apoptosis process [24,25]. Previous studies indicated that ROS accumulation occurring during many virus infections might cause cellular damage and apoptosis [26,27,28]. However, ROS are generated as a result of IBV infection in chicken embryo kidney cells. To further study the relation between IBV and oxidative stress in the chicken macrophage HD11 cell line, during the phases of infection, we looked into how directly a viral infection affected the host cells’ oxidative stress. The findings demonstrate that ROS formation can be caused by IBV infection.

Mitochondria are considered oxidative damage sensors and have a key role in apoptosis. Excessive ROS can trigger the oxidation of intracellular proteins and induce DNA injury to shorten telomeres, followed by further activating apoptosis-related signals [29]. Loss of selective ion permeability can indicate intrinsic apoptosis, triggering mitochondrial permeability transition pore production and apoptosis-initiating factor release, which then activate caspases [30]. Many viral infections may lead to ROS accumulation and induce mitochondrial cascading [31]. Across swine testicle (ST) cells, PPV infection activated ROS buildup and mitochondria-mediated apoptosis [19]. During infection with TGEV, oxidative stress may change the intracellular processes, which results in apoptosis [20]. There are relatively few works on the variations in membrane potential in IBV-infected host cells. Thus, our results suggest that Δψm was significantly lowered in a time-dependent manner within the IBV-infected HD11 cells, and IBV-induced ROS accumulation led to mitochondrial outer membrane permeabilization.

Oxidative stress is widely recognized as an activator for apoptosis, which is induced by a variety of stimuli, such as viral infection. ROS are critical in many examples of virus-induced apoptosis [32]. Japanese encephalitis virus (JEV) replication enhanced intracellular ROS production, activated ASK1-ERK/p38 MAPK signaling, and caused time-dependent apoptosis in human promonocyte cells [33]. Porcine reproductive and respiratory syndrome virus (PRRSV) releases cytochrome C via ROS accumulation, which further induces apoptosis through activation of the mitochondrial pathway [34]. Increased ROS levels were associated with the triggering of NF-κB (p65) and p53 responses after Rift Valley fever virus (RVFV) infection [35]. Our past research demonstrated that IBV infection can induce apoptosis in HD11 cells via both intrinsic and extrinsic mechanisms [8]. Caspase-3 is a cysteine-aspartic protease which plays a crucial role in the process of apoptosis and is widely used as a biomarker for apoptosis. The mitochondrial apoptotic signal in the apoptotic pathway is mainly controlled by the Bcl-2 protein family, primarily including the Bcl-2 and Bax proteins. Bcl-2 and Bax proteins are located upstream of the mitochondria and are important regulatory factors for changes in mitochondrial membrane permeability, also participating in the regulation of caspase-3 activity [24]. To study whether oxidative stress is implicated in IBV-induced apoptosis, we studied ROS’s impact on cell apoptosis. Our studies indicate that two ROS scavengers (PDTC and NAC) significantly suppressed the reduction in Bcl-2, caspase-3 activation, apoptosis occurrence, and the increase in Bax induced by IBV infection, indicating that ROS are crucial to IBV-induced cellular apoptosis.

The initiation of ROS affects virus replication in both directions. On the one hand, the purpose of ROS is to protect the cell from invasive viral infections. On the other side, certain virus replication types benefit from the generation of ROS [36]. When it comes to herpesviruses, ROS not only promote virus replication but also cause the virus to reactivate from dormancy and might be a factor in virally induced tumors [37]. In addition, Japanese encephalitis virus (JEV) replication-incompetent virions produced by short-wavelength ultraviolet (UV) irradiation may also cause host cell death [38]. To investigate whether the activation of ROS has an effect on IBV replication in HD11 cells, we used two blockers to inhibit the accumulation of ROS. After being treated with antioxidants, the viral titers were reduced, and the cell viability was significantly increased after virus infection in HD11 cells. These findings show that virus replication is dependent on ROS signal activation. Furthermore, the production of ROS is also affected by the replication of some viruses. This demonstrates that IBV-induced ROS generation in HD11 cells is dependent on viral replication, as UV-inactivated IBV or NH_4_Cl inhibited ROS generation. As a whole, viral replication and the accumulation of ROS have an interplay with each other.

Several viral proteins have been shown to cause the accumulation of reactive oxygen species and the activation of apoptosis in host cells. For instance, ORF3a, a SARS-CoV-2 accessory viral protein, induces apoptosis in host cells and suppresses their defense mechanisms [39]. In addition, the HN protein of Newcastle disease virus, through the induction of lysosomal membrane permeabilization, induces cell apoptosis [40]. In the past, the study of the IBV N protein mainly focuses on modulation of the immune signaling pathway [41]. However, it is unclear whether the N protein plays a dominant role in cell apoptosis. In this study, we preliminarily investigated its function in the modulation of apoptosis and demonstrated that the IBV N protein is capable of accumulating ROS and inducing apoptosis in the transfected HD11 cells, which might be important for the pathogenesis of IBV. In this study, we highlighted the significance of the N protein in relation to ROS accumulation and apoptosis induction. However, it is essential to recognize that IBV comprises other components, such as the spike (S) protein, membrane (M) protein, and other proteins, which may also contribute to these processes. Although our current research did not encompass an analysis of these additional components, they may also be involved in apoptosis and oxidative stress induction. Subsequent studies will expand our understanding by exploring the role of these factors in pathogenesis, leading to a comprehensive understanding of the effects of IBV on apoptosis mechanisms.

## 5. Conclusions

In conclusion, the findings show that HD11 cells exposed to IBV produce an accumulation of ROS. By activating mitochondria-mediated apoptotic signaling, oxidative stress further increased cell apoptosis. Furthermore, ROS activation was required for IBV replication, which declined when ROS activation was prevented. Expression of the IBV-N gene can cause ROS accumulation and cell apoptosis in HD11 cells. Our findings might provide an understanding of how IBV and the host interact as well as the pathogenesis of IBV infection.

## Figures and Tables

**Table 1:** Sequences of chicken primer pairs used for qRT-PCR.

Gene	Forward Primer (5′–3′)	Reverse Primer (5′–3′)
Bax	GGTGACAGGGATCGTCACAG	TAGGCCAGGAACAGGGTGAA
Bcl-2	TGTTTCTCAAACCAGACACCAA	CAGTAGGCACCTGTGAGATCG
IBV-N	AAGCTTATGGCAAGCGGTAAAGCAGCT	GAATTCTCAAAGTTCATTCTCTCCTAGAGCTGC
β-actin	TGCTGTGTTCCCATCTATCG	TTGGTGACAATACCGTGTTCA

## Data Availability

The data used to support the findings of this study are available from the corresponding author upon request.
